# Yin and yang of interleukin-17 in host immunity to infection

**DOI:** 10.12688/f1000research.10862.1

**Published:** 2017-05-23

**Authors:** Shibali Das, Shabaana Khader

**Affiliations:** 1 Department of Molecular Microbiology, Washington University in St. Louis, St Louis, MO, USA

**Keywords:** IL-17, host immune response, pathogens

## Abstract

The interleukin-17 (IL-17) family cytokines, such as IL-17A and IL-17F, play important protective roles in host immune response to a variety of infections such as bacterial, fungal, parasitic, and viral. The IL-17R signaling and downstream pathways mediate induction of proinflammatory molecules which participate in control of these pathogens. However, the production of IL-17 can also mediate pathology and inflammation associated with infections. In this review, we will discuss the yin-and-yang roles of IL-17 in host immunity to pathogens.

## Introduction

The interleukin-17 (IL-17) cytokine family is composed of six defined members, including IL-17A through IL-17F ^1^. Among the IL-17 family members, IL-17A and IL-17F have the best-characterized proinflammatory activity. Although the genes encoding IL-17A and IL-17F are both located on chromosome 1 and 6 (respectively), in mice and humans ^2^, their functions can be similar or distinct, depending on the type of infection ^3^. Although other members of the IL-17 family such as IL-17B, IL-17C, and IL-17D can also induce the production of proinflammatory cytokines and chemokines ^4^, their functions are not as well characterized and will be only briefly summarized. The IL-17 cytokine family employs various cytokine receptors (IL-17RA, IL-17RB, IL-17RC, IL-17RD, and IL-17RE) on target cells to mediate their biological functions ^5^. IL-17R is a heteromeric receptor comprising IL-17RA and IL-17RC and mediates signaling of IL-17A and IL-17F. In contrast, partnering of IL-17RA with IL-17RB is thought to mediate IL-17E signaling whereas IL-17RA partnering with IL-17RE mediates IL-17C signaling ^5^. IL-17Rs are ubiquitously expressed in various cell types ranging from leukocytes to fibroblasts, epithelial cells, mesothelial cells, endothelial cells, and keratinocytes ^6, 7^. IL-17A or IL-17F mediates their biological function through the IL-17R via the activation of nuclear factor-kappa B (NF-κB) and mitogen-activated protein kinase (MAPK), leading to the production of proinflammatory cytokines and chemokines ^8– 11^. Tumor necrosis factor receptor-associated factor 6 (TRAF-6) plays an indispensable role in IL-17R signaling as IL-17 stimulation fails to activate IL-17R signaling in TRAF-6-deficient mouse embryonic fibroblasts ^12, 13^. In addition, NF-κB activator 1 (Act1) is important for IL-17R signaling, where it acts as an adapter molecule for the recruitment of TRAF-6 with IL-17R ^14, 15^. In this review, we will use IL-17 to refer to IL-17A.

Upon exposure to pathogen or pathogen-associated molecular patterns (PAMPs), dendritic cells, monocytes, and macrophages induce cytokines such as IL-23, IL-1β, IL-6, and transforming growth factor-beta (TGF-β), which initiate the differentiation and polarization of naïve CD4 ^+^ T cells toward the T helper cell type 17 (Th17) subsets ^16^. Low levels of TGF-β support induction of the transcription factor RAR-related orphan receptor gamma (RORγ) and differentiation toward a Th17 subset, while high levels of TGF-β along with defined cytokines such as IL-2 mediate transition to regulatory T cells (Tregs) through the activation of the transcription factor, fork head box P3 (Foxp3) ^17– 19^. Th17 cells are considered a primary source of IL-17 and co-produce other cytokines, including IL-22, IL-21, tumor necrosis factor-alpha (TNF-α), and granulocyte macrophage-colony-stimulating factor (GM-CSF) ^20, 21^. However, depending on the cytokine milieu, Th17 cells can exhibit substantial plasticity in cytokine production ^22^. Th17 cells can also co-express GATA binding protein 3 (GATA-3) or T-box transcription factor (T-bet), allowing them to progress into either IL-4-expressing or interferon-gamma (IFN-γ)-expressing Th17 subsets ^23^. Thus, it is likely that during infection *in vivo*, Th17 cells exhibit substantial plasticity and can co-express Th17 cytokines along with other Th1, Th2, and Treg-associated cytokines. Additionally, in response to early IL-23 and IL-1β production by myeloid cells, innate cells such as γδ T cells ^24^ and group 3 innate lymphoid cells (iLC3) ^25, 26^ can produce IL-17 and mediate early immune responses. Other immune cells such as neutrophils ^27– 29^, invariant natural killer T (iNKT) ^30^ cells, innate Th17 cells (iTh17) ^31^, and natural killer (NK) ^32^ cells can also produce IL-17 through stimulation of TGF-β, IL-1β, IL-6, IL-23, or alpha-galactoceramide (α-galcer) ^33^. A primary mechanism by which IL-17 mediates protection against pathogens (such as *Klebsiella*, *Candida*, and *Chlamydia*) is through the induction of chemokines and cytokines and downstream recruitment of neutrophils ^34, 35^. IL-17 can act alone or in synergy with other cytokines such as TNF-α and IL-22 to mediate induction of neutrophil-recruiting chemokines such as granulocyte-colony-stimulating factor (G-CSF) and C-X-C motif chemokine ligand 1 (CXCL1) and regulate neutrophil-mediated destruction of pathogens ^36^. In addition, IL-17 alone or synergistically with IL-22 or 1,25-dihydroxyvitamin D3 induces the expression of anti-microbial proteins such as Lipocalin-2 ^37^, β-defensin ^38^, S100A7 (psoriasin), S100A8/9 (calprotectin), and cathelicidin (LL37), resulting in pathogen control ^11^, likely through direct anti-microbial actions. Our recent knowledge on the role of IL-17 in immunity to various pathogens, including extracellular ^39, 40^ or intracellular ^41– 43^ bacteria, fungi ^9, 44^, viruses ^45^, and parasites ^46, 47^, has emerged within the past decade. In this short review, we will summarize the recent progress in the field of IL-17-mediated immune responses against various infections.

## Role of IL-17 in immunity to extracellular bacterial infection

The role of IL-17 in host defense against extracellular bacteria is thought to be primarily through the induction of anti-microbial molecules and mediation of neutrophil recruitment at the site of infection guided by chemokine gradients. Early studies with IL-17R-deficient mice demonstrated a critical role for IL-17 in the clearance of the extracellular pulmonary pathogen *Klebsiella pneumoniae* infection. IL-17R-deficient mice upon infection with *K. pneumoniae* produced lower levels of the neutrophil-driving cytokine G-CSF and neutrophil-recruiting chemokine, macrophage inflammatory protein-2 (MIP-2). These changes in cytokines and chemokines in IL-17R-deficient mice resulted in decreased neutrophil infiltration into the lung and subsequently higher bacterial burden along with increased mortality ^48^. Additionally, IL-17R-deficient mice are more susceptible to a variety of mucosal extracellular pathogens, including the gut-specific pathogen *Citrobacter rodentium* ^49^, skin pathogen *Staphylococcus aureus* ^50^, and pulmonary pathogen *Bordetella pertussis* ^51^. Moreover, neutralization of IL-17 resulted in the suppression of anti-microbial peptide β-defensin production, which killed invading *S. aureus* at mucosal surfaces ^52^. These studies provide the consensus that upon infection with extracellular pathogens, γδ T cells ^53^, iLC3 ^54^, and iNKT ^55^ cells are important early producers of IL-17 which are associated with innate immunity following extracellular bacterial infections. In addition, Th17 cells are involved in the IL-17-mediated responses associated with adaptive immune responses ^56, 57^. Therefore, these studies suggest that induction of IL-17 and synchronized production of anti-microbial molecules and neutrophil recruitment help the resolution of extracellular infection. During extracellular pathogenesis, the major IL-17 responsive cell population is thought to be mucosal epithelial cells ^58, 59^. However, other studies suggest that macrophage or dendritic cells (or both) also express IL-17R and respond to IL-17 and downstream protective responses ^4, 54^. Recently, it was reported that innate immune defense against a highly antibiotic-resistant strain of *K. pneumoniae* depends on crosstalk between inflammatory monocytes and innate lymphocytes which is mediated by TNF-α and IL-17 ^54^. IL-17-producing resident epidermal γδ T cells are essential for protecting the host against a subsequent staphylococcal infection ^60^. IL-17-dependent neutrophil-mediated protection is also observed during spontaneous *S. aureus* infection ^61, 62^ and *K. pneumoniae* infection ^63– 65^. Although in most studies IL-17 plays a protective role during extracellular bacterial infections, in some cases IL-17 can also mediate pathology associated with the infection. For example, the periodontal extracellular bacteria *Porphyromonas gingivalis* can directly promote autoimmune arthritis by the induction of Toll-like receptor 2 (TLR2)/IL-1Rα-driven IL-17 response in DBA/1J mice ^66^. Furthermore, increased frequency of IL-17 ^+^ cells was observed in gingival tissue of patients with periodontitis ^67^, likely produced by human CD4 ^+^ T cells ^68^. Similarly, *B. pertussis* infection can bias the host immune response toward IL-17 production, which may be associated with cough pathology in pertussis infection ^56, 69^. Additionally, IL-17 is associated with the neutrophilia and airway inflammation during *Haemophilus influenza* infection in mice undergoing allergic airway disease ^70^. Thus, IL-17 has an important role in protective immunity to extracellular pathogens through release of anti-microbial proteins from cell types such as epithelial cells and neutrophils (and monocytes). On the other hand, IL-17 induced in response to infection may mediate excessive inflammation and pathology.

## Role of IL-17 in intracellular bacterial infection

Although infection by intracellular bacteria is predominantly cleared by Th1 immune responses, recent studies have described an emerging role for IL-17 in protection against intracellular pathogens such as *Listeria monocytogenes* ^71^, *Mycoplasma pneumonia* ^72^, *Legionella pneumophila* ^73, 74^, *Salmonella typhimurium* ^75^, *Chlamydia muridarum* ^76^, *Francisella tularensis* ^77^, and *Mycobacterium tuberculosis* ^78^. Following infection with intracellular pathogens, like infection with extracellular pathogens, both innate cells such as iLC-3 ^64^ and γδ T cells ^79^ and adaptive cells such as Th17 cells ^80^ are the primary producers of IL-17. But during intracellular infection, unlike extracellular infection, macrophages or myeloid cells have been shown to be major responder cells to IL-17. In response to IL-17 stimulation, macrophages and myeloid cells secrete higher amounts of anti-microbial cytokines such as TNF-α, IFN-γ, or IL-12 and contribute to host immune response against infections such as *F. tularensis* ^77^. Although γδ T cell-derived IL-17 has played a more prominent role in *L. monocytogenes* ^41^, *M. tuberculosis* ^81^, *F. tularensis* ^82^, and *Mycobacterium bovis* Bacillus Calmette-Guérin ^83^ infections, Th17 cells as well as CD8 ^+^ cells are also involved in the antigen-specific production of IL-17 at the site of infection ^84^. In addition, IL-17-deficient mice experience higher bacterial burden associated with disorganized granuloma formation (reduced monocyte, granulocyte, and T cell recruitment within the granuloma) during infections with intracellular pathogens such as *F. tularensis* ^77^, *S. typhimurium* ^85^, or *M. tuberculosis* ^86^. In some infection models, including *C. muridarum*, IL-17 complemented the protective role imparted by the IL-12/IFN-γ axis through the involvement of myeloid differentiation factor 88 (MyD88) signaling where MyD88-deficient infected mice showed reduced IL-17 responses along with reduced neutrophil infiltration, which is important for early control of disease pathogenesis ^87, 88^. However, excess IL-17 production is detrimental for the host, as IL-10-deficient mice exhibit increased mortality after pulmonary *F. tularensis* infection due to excessive inflammation induced by IL-17 ^89^, which suggests that IL-17 is tightly regulated by IL-10. However, other evidence suggests that the contribution of IL-17 may serve a more compensatory function under unfavorable conditions such as in the absence of type I and II interferon signaling, where a low-magnitude IL-17 response to *L. monocytogenes or M. tuberculosis* infection is evident ^87, 90^. On the contrary, early studies suggest that IL-17-mediated immunity is dispensable against *M. tuberculosis* infection as evident by the results obtained from either anti-IL-17 treated or IL-17R-deficient mice which were not more susceptible against infection with less virulent lab-adapted *M. tuberculosis* strains as compared with wild-type mice ^91, 92^. However, the involvement of IL-17 in mucosal vaccine-driven protection in murine models of tuberculosis seems to be crucial, as suggested by Gopal *et al*. ^93^. IL-17-mediated induction of CXCL-9-11 is responsible for the recruitment of protective antigen-specific T cells as well as induction of CXCL-13 to localize C-X-C motif chemokine receptor 5 (CXCR5)-positive cytokine-producing T cells within lung granulomas of *M. tuberculosis*-infected mice ^94^. Interestingly, IL-17 responses were involved in protection against a hyper-virulent clinical isolate *M. tuberculosis* HN878 strain, as IL-17-deficient mice infected with *M. tuberculosis* HN878 had significantly higher bacterial burden along with reduced chemokine expression and less organized granuloma formation ^95^. However, there are some contradictory views regarding the role of IL-17 in the context of human tuberculosis. Some studies support the protective role of IL-17 during human tuberculosis as IL-17 helps in the generation of proinflammatory cytokines such as IL-12 and IFN-γ and restricts pathogenesis within the host ^96^. In contrast, other reports identified that IL-17 had a negative correlation with tuberculosis treatment and disease outcome ^97^. In addition, IL-17-producing T cells are reported to play an immunopathological role in patients with multidrug-resistant *M. tuberculosis* by promoting severe tissue damage, which may be associated with low effectiveness of the second-line drugs employed during treatment ^97^. Moreover, IL-23-dependent IL-17 production is associated with neutrophil accumulation and inflammation during a chronic re-stimulation model of tuberculosis ^98^. Indeed, exacerbated production of IL-17 appears to drive pathology by inducing S100A8/A9 proteins that recruit neutrophils into the lung ^99^ and cause excessive inflammation in mice during tuberculosis. Therefore, at least in the context of tuberculosis, the *M. tuberculosis* strain to some extent specifically dictates the protective role of IL-17. Therefore, during intracellular pathogen infections, although IL-17 is mostly associated with host protection through regulation of chemokine and cytokine balance and infiltration of different immune cells to the site of infection, IL-17 activity should be tightly regulated in order to maintain the fine balance between protection and pathology induced by IL-17.

## Role of IL-17 during sepsis

Although sepsis is a syndrome rather than a disease itself, the role for IL-17 in experimental murine sepsis models and human sepsis has been studied. In a colitis model, both IL-17-deficient mice and mice treated with IL-17 neutralizing antibody resulted in significant improvement in survival which was associated with reduced disease pathology and decreased bacteremia ^100, 101^. In line with this observation, IL-17 also drives sepsis-associated acute kidney injury by increasing the levels of proinflammatory cytokines and inducing neutrophil accumulation and tubular epithelial cell apoptosis ^102^ in mouse models. More recently, targeting IL-17 has been shown to attenuate IL-18-dependent disease severity in a neonatal sepsis mouse model ^103^. *In vitro* studies with the peripheral blood mononuclear cells (PBMCs) from healthy donors and patients undergoing severe sepsis showed increased Th17 cells in patients with sepsis when compared with healthy donors. Additionally, IL-17 neutralization increased IL-10 production in PBMCs, suggesting a role for IL-10 in modulating immune responses during sepsis ^104^. Thus, IL-17 has a pathological role in sepsis, and targeting IL-17 may serve to resolve sepsis and sepsis-induced pathogenesis.

## Role of IL-17 in parasitic infection

Although IL-17 has been considered an important player in the mediation of host protection against extracellular and some intracellular pathogens, the role of IL-17 in host defense against intracellular protozoan parasites remains less well studied. Infection studies demonstrate that Th17 cells mediate host defense against *Trypanosoma cruzi* ^105^, *Toxoplasma gondii* ^106^, *Leishmania braziliensis* ^107^, and *Echinococcus granulosus* ^108^ infections. NK cells are a major source of IL-17 during toxoplasmosis ^32^. In addition, CD4 ^+^ and CD8 ^+^ cells express IL-17 in human toxoplasmosis and impact human pregnancy by controlling parasite invasion and replication which often cause fetal malfunction or abortion ^109^. Increased IL-17 levels were detected in the PBMCs and tissue from leishmaniasis-infected patients and associated with enhanced neutrophil and macrophage-mediated destruction of the parasite ^110^. Furthermore, IL-17R-deficient mice were associated with reduced production of the chemokine MIP-2 along with the suboptimal levels of neutrophil recruitment and higher parasitic load as compared with wild-type counterparts ^111^. Additionally, during echinococcosis, IL-17 plays a crucial immune protective role by regulating the Tregs which are associated with tolerance during infection ^112^. In contrast, in human cutaneous leishmaniasis ^113– 115^ and *Eimeria tenella* infection in chickens ^116^, IL-17 contributed to the pathology through excessive inflammation and subsequent tissue damage. A recent report suggests that *Leishmania guyanensis* is associated with a cytoplasmic virus which enhances parasite virulence and is linked to increased IL-17 levels induced following *L. guyanensis* infection ^115^. Neutralization of IL-17 was effective in reducing disease severity in a mouse model of cutaneous leishmaniasis, suggesting that IL-17 may have a strain-specific immunological role during leishmaniasis infection ^115^. Despite having a protective role against *T. gondii* infection, IL-17 had a deleterious effect that is evident where neutralization of IL-17 had a partial protective role against the fatal disease ^117^, through co-production of IL-10 and IFN-γ which regulated the exacerbated inflammation induced by IL-17. Taken together, these reports argue with previous reports and present new evidence in favor of the pathological role of IL-17 during parasitic infections. Therefore, during parasitic infection, the role of IL-17, whether protective or pathologic, has yet to be firmly established.

## Role of IL-17 in fungal infection

IL-17 plays an immunologically important host protective role against fungal pathogens such as *Candida albicans* ^118^, *Cryptococcus neoformans* ^119^, *Pneumocystis carinii* ^120^, and *Aspergillus fumigatus* ^121^ in both humans and mice. Similar to the mechanisms seen in the intracellular and extracellular bacterial infections, fungal pathogens elicit IL-17 protective effects through the release of proinflammatory cytokines, chemokines, and anti-microbial peptides. During infection, IL-17 is expressed by various cell types, including oral resident γδ T cells ^122^, iLC3 ^123^, and natural Th17 cells ^122^. Moreover, the IL-17 cytokine family contributes in the development of NK cells which promote anti-fungal immunity by secreting GM-CSF, necessary for the fungicidal activity of neutrophils ^124, 125^. Recent advances in the field of oral candidiasis depict oral epithelial cells (OECs) as the major responder cells to IL-17 signaling ^126^. These OECs produce β-defensin 3 through IL-17R signaling which is necessary for protection against oral candidiasis through both a neutrophil-dependent and -independent manner ^118^. Caspase recruitment domain family member 9 (CARD-9) signaling is associated with the production of IL-17 during fungal infections ^127^. Accordingly, humans with CARD-9 ^128^ or IL-17R deficiency have increased mucocandidiasis ^129^ and are more vulnerable during systemic candidiasis ^124^, and decreased IL-17 production is associated with increased susceptibility to fungal pathogens ^130^. These studies suggest that fungal pathogens are dependent on IL-17-mediated recruitment of inflammatory cells for fungal control. In contrast, IL-17C subset is associated with lethal inflammation during candidiasis through induction of proinflammatory cytokines in renal epithelial cells ^131^. Moreover, the IL-23/IL-17 pathway promotes inflammation and susceptibility to fungal infectious disease models such as *C. albicans* and *A. fumigatus* through excessive inflammation, which impairs anti-fungal resistance against those infections ^132– 134^. Therefore, critical observation on the particular role played by the IL-17 cytokine family is necessary before considering IL-17 signaling as a potential drug target.

## Role of IL-17 in viral infection

Recent studies have addressed whether IL-17 is protective or pathologic in response to viral infections such as influenza (H1N1, H5N1), vaccinia virus, Epstein-Barr virus (EBV), herpes simplex virus (HSV), respiratory syncytial virus (RSV), human immunodeficiency virus (HIV), and hepatitis (B and C). Although several studies have suggested a protective role imparted by IL-17 signaling in host immunity during influenza infection, other studies have suggested a more pathological role instead. For example, it has been observed that depletion of IL-17 resulted in a more severe disease outcome in a mouse model of influenza, which was associated with increased weight loss as well as reduced survival ^135, 136^. Furthermore, adoptive transfer of Th17 polarized antigen-specific effector cells has been shown to be protective in mice challenged with a lethal dose of influenza, thus suggesting a protective role for IL-17 that is independent of IFN-γ ^137^. In contrast, IL-17R-deficient mice have also been shown to have reduced neutrophil influx and decreased inflammation, suggesting a pathological role for IL-17 during influenza challenge ^138, 139^. The genetic background of mice used and the influenza dose used were different between the studies, suggesting a protective or pathological role for IL-17 in influenza. Therefore, these studies suggest that the genetic background and infectious dose may act as a determining factor regarding the protective or pathologic role of IL-17 during influenza infection. In contrast, IL-17 is associated with the pathology in 2009 pandemic influenza A (H1N1)-induced acute lung injury ^140^. Additionally, IL-17 levels are associated with the exacerbated disease pathology induced following viral infections such as hepatitis ^141, 142^, vaccinia virus ^143, 144^, RSV ^145– 147^, HSV ^148, 149^, and EBV ^150, 151^. During viral infections (such as hepatitis), IL-17 can either potentiate early neutrophil infiltration at the site of infection ^152^ or inhibit NK cell-mediated host immune response (for example, vaccinia virus infection) ^153^. Neutralization of IL-17 not only reduced the disease severity but also reduced the viral load in the host and improved survival of the host during HSV ^72, 148^ and Dengue virus ^154^ infections. Despite having a pathological role against most viral infections, IL-17 was suggested in several reports to have a protective role during HIV infection. Along with the Th17 cells, a subset of CD8 ^+^ cells which produce IL-17, also known as TC17, are important in the context of viral infection, although the detailed role of TC17 has yet to be delineated ^155, 156^. Moreover, Treg/Th17 ratios dictate the outcome of infection as well as effectiveness of anti-retroviral treatment ^157, 158^. Therefore, the balance between the Treg and Th17/Tc17 is suggested to be more important than that of the expression of IL-17 alone ^159^. However, some recent data also suggest that during HIV infection IL-17 levels have a negative correlation with HIV plasma viral load ^160^. Therefore, these data together suggest that IL-17 may be contributing to the inflammatory injury in response to viral infection, but the recruitment of inflammatory cells such as neutrophils or lymphocytes may be required for protection. We propose that the full array of IL-17 responses during various viral infections has yet to be fully delineated.

## Anti-IL-17 therapies and impact on host immunity to infections

Exacerbated IL-17 production is linked to excessive inflammation-associated complications such as autoimmunity, chronic obstructive pulmonary disease (COPD), and contact dermatitis. Moreover, *P. gingivalis* infection predisposes the patient to the potential risk of acquiring autoimmune disorders, specifically rheumatoid arthritis (RA) ^161, 162^ through excessive inflammation (induced by IL-17) or generation of autoantibodies. As a result, diseases such as psoriasis ^163^, RA ^164^, and contact dermatitis ^165^ are emerging as particularly strong IL-17-driven disorders. Similarly, excessive IL-17 leads to the upregulation of neutrophil-attracting chemokines and subsequent neutrophil infiltration and inflammation during COPD ^166, 167^. A number of biologic drugs targeting IL-17A/F and IL-17RA are being used or evaluated as treatment options against several diseases, such as COPD ^168^, psoriasis, and RA, with impressive efficacy ^169, 170^. However, IL-17 is strongly associated with the protection against *Mtb* clinical isolates and fungal infections. IL-17 and IL-17RA single-nucleotide polymorphisms enhance the risk of fungal diseases such as candidiasis ^171^ and bacterial disease such as pulmonary tuberculosis in certain cohorts ^172, 173^. Moreover, deficiency in CARD-9 ^174^ or gain of function of signal transducer and activator of transcription 1 (STAT-1) ^175^ impairs IL-17 signaling and these mutations are associated with the chronic candidiasis. Therefore, we suggest that anti-IL-17 treatments may have a detrimental effect on the overall immunity of those individuals as they may become immunocompromised, resulting in predisposition toward the risk of acquiring several infections (including *Candida* ^176^ and *Mycobacterium* ^177^).

## Conclusions

**Figure 1.**
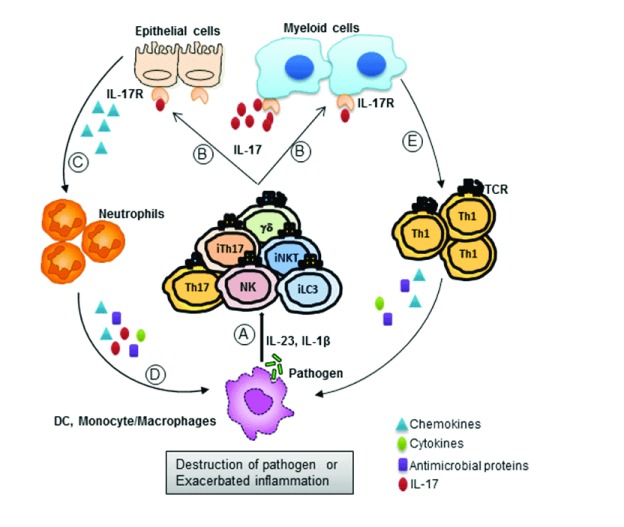
Yin-and-yang roles of IL-17 during infections. As the host immune system encounters a pathogen, host immune cells respond by releasing an array of cytokines such as IL-23, IL-6, and IL-1β. ( **A**) These cytokines elicit IL-17 production from both innate cells (iLC3, NK, iNKT, iTH17, and γδ T) and adaptive cells (Th17 and Tc17). ( **B**) This IL-17 then acts on responder cells, which express IL-17Rs on the cell surface, such as epithelial cells or myeloid cells. ( **C**) Through IL-17R signaling, these responder cells produce chemokines which help recruit neutrophils to the site of infection. ( **D**) These recruited neutrophils destroy the pathogen (mostly extracellular) through the production of cytokines, chemokines, and anti-microbial peptides. ( **E**) Similarly, myeloid cells are also able to restrict pathogen establishment through activation and recruitment of Th1 cells. These Th1 cells secrete proinflammatory cytokines, chemokines, and anti-microbial peptides to restrict pathogenesis. On the other hand, excessive inflammation at the site of infection may lead to exacerbated disease pathology. IL, interleukin; IL-17R, interleukin 17 receptor; iLC3, group 3 innate lymphoid cell; iNKT, invariant natural killer T; iTH17, innate T helper cell type 17 cell; NK, natural killer; Th, T helper cell type.

The importance of IL-17 in different infectious models is now well established. Although there are several infections where the role of IL-17 is not clear, IL-17 plays distinct yin-and-yang roles in a majority of the cases. IL-17 plays a protective role against the infection, and excess IL-17 promotes pathology and tissue destruction. The overall global role for the involvement of IL-17 in infection models is summarized in Figure 1 and Table 1. Upon exposure to pathogens (bacteria, fungus, or virus), myeloid cells produce factors that promote the production of IL-17 from both innate and adaptive cells. IL-17 then acts on primary responder cells (epithelial, macrophage, or myeloid cells), thereby inducing the production of other anti-microbial peptides, chemokines, and cytokines. IL-17-induced chemokines recruit neutrophils (and other immune cells) to the site of infection and restrict pathogenesis. On the other hand, this pathway can mediate excessive inflammation and exacerbated pathology at the infectious milieu. Hence, careful observation on the role of IL-17 is necessary to improve the overall treatment strategy against such infections. Therefore, it is important to critically consider the yin-and-yang roles of IL-17 while designing novel strategies to target specific pathways for control of pathogens.

**Table 1.** Description of infections where protective or pathologic roles of IL-17 have been demonstrated.

	Protective roles of IL-17	Pathologic role of IL-17
Extracellular bacteria	*Klebsiella pneumoniae* ^48^, *Citrobacter rodentium* ^49^, *Staphylococcus aureus* ^50^, and *Bordetella pertussis* ^51^	*Bordetella pertussis* ^56, 69^, *Porphyromonas gingivalis* ^66^, and *Haemophilus influenza* ^70^
Intracellular bacteria	*Listeria monocytogenes* ^71^, *Mycoplasma pulmonis* ^72^, *Legionella pneumophila* ^73, 74^, *Salmonella typhimurium* ^75^, *Chlamydia muridarum* ^76^, *Francisella tularensis* ^77^, and *Mycobacterium tuberculosis* ^78^	*Mycobacterium tuberculosis* ^96– 98^
Parasites	*Trypanosoma cruzi* ^104^, *Toxoplasma gondii* ^105^, *Leishmania braziliensis* ^106^, and *Echinococcus* *granulosus* ^107^	*Leishmania major* ^112, 113^, *Leishmania guyanensis* ^114^, *Eimeria tenella* ^115^, and *Toxoplasma gondii* ^116^
Fungus	*Candida albicans* ^117^, *Cryptococcus neoformans* ^118^, *Pneumocystis carinii* ^119^, and *Aspergillus fumigatus* ^120^	*Candida albicans* ^130– 133^ and *Aspergillus fumigatus* ^133^
Virus	H5N1 ^134– 136^ and HIV ^154, 155^	H1N1 ^137– 139^, respiratory syncytial virus ^144– 146^, herpes simplex virus ^147, 148^, Epstein-Barr virus ^149, 150^, vaccinia virus ^142, 143^, Dengue virus ^153^, hepatitis B and C virus ^140, 141^, and HIV ^159^

HIV, human immunodeficiency virus; IL-17, interleukin-17.

## Abbreviations

CARD-9, caspase recruitment domain family member 9; COPD, chronic obstructive pulmonary disease; CXCL, C-X-C motif chemokine ligand; EBV, Epstein-Barr virus; G-CSF, granulocyte-colony-stimulating factor; GM-CSF, granulocyte macrophage-colony-stimulating factor; HIV, human immunodeficiency virus; HSV, herpes simplex virus; IFN-γ, interferon-gamma; IL, interleukin; IL-17R, interleukin 17 receptor; iLC3, group 3 innate lymphoid cell; iNKT, invariant natural killer T; MIP-2, macrophage inflammatory protein 2; MyD88, myeloid differentiation factor 88; NF-κB, nuclear factor-kappa B; NK, natural killer; OEC, oral epithelial cell; PBMC, peripheral blood mononuclear cell; RA, rheumatoid arthritis; RSV, respiratory syncytial virus; TGF-β, transforming growth factor-beta; Th17, T helper cell type 17; TNF-α, tumor necrosis factor-alpha; TRAF-6, tumor necrosis factor receptor-associated factor 6; Treg, regulatory T cell.
